# Comparative testis proteome dataset between cattleyak and yak

**DOI:** 10.1016/j.dib.2016.05.071

**Published:** 2016-06-03

**Authors:** Fang Yang, TserangDonko Mipam, Lei Sun, Shumin Yu, Xin Cai

**Affiliations:** a College of Veterinary Medicine, Sichuan Agricultural University, Chengdu 611130, Sichuan, China; b College of Life Science and Technology, Southwest University for Nationalities, Chengdu 610041, Sichuan, China; c School of Life Science and Engineering, Southwest University of Science and Technology, Mianyang 621010, Sichuan, China

## Abstract

Cattleyak are hybrid between cattle and yak, which exhibit equivalent adaptability on the Qinghai-Tibetan Plateau as yak and much higher capability in economic traits. However, the F1 males of cattleyak are infertile due to spermatogenic arrest and this greatly restricts the effective utilization of this hybrid. In this data article, differentially expressed proteins (DEPs) were identified from testis proteome of cattleyak and yak using high-performance liquid chromatography–electrospray tandem mass spectrometry (LC–ESI-MS/MS). All the DEPs were subjected to functional classification by Gene Ontology (GO) analysis and gene-pathway annotation by Kyoto Encyclopedia of Genes and Genomes (KEGG). The comparative testis proteome dataset here can shed new light on the molecular characteristics of male infertility of cattleyak on proteome level, “Comparative iTRAQ proteomics revealed proteins associated with spermatogenic arrest of cattleyak” [1].

**Specifications Table**

**Table:** 

Subject area	Biology
More specific subject area	Reproductive biology
Type of data	Table, figure
How data was acquired	Mass spectroscopy, data acquired by LC–ESI-MS/MS analysis based on Triple TOF 5600
Data format	Analyzed
Experimental factors	Testis of each animal was obtained by veterinary surgical operation. Fat and fascia tissues surrounding the testis were eliminated and were snap frozen in liquid nitrogen (−196 °C), transported to laboratory and stored at −80 °C until analysis.
Experimental features	Total protein was taken out of each sample solution and was digested with Trypsin Gold (Promega, Madison, WI, USA). After trypsin digestion, peptides were dried by vacuum centrifugation and then reconstituted and processed according to the manufacture׳s protocol for 8-plex iTRAQ reagent (Applied Biosystems). SCX chromatography was performed with a LC-20AB HPLC Pump system (Shimadzu, Kyoto, Japan). Data acquisition was performed with a TripleTOF 5600 System (AB SCIEX, Concord, ON).
Data source location	Mianyang, Sichuan, China
Data accessibility	The data is available with this article

**Value of the data**

• This is the first dataset to examine the male infertility of cattleyak by comparative testis proteome.
• The dataset lays the basis for a clear presentation of up-regulated proteins in cattleyak associated with various stresses/cell adhesion/germ cell migration and down-regulated proteins associated with defects in various metabolic processes/cellular processes during spermatogenesis.
• The DEPs indicate their potential functions in spermatogenic arrest in cattleyak.

## 1 Data

This data is related to DEPs from testis proteome of cattleyak and yak using LC–ESI-MS/MS [1]. All the DEPs were categorized by GO and KEGG analysis. See Supplementary Figures and Tables provided.

## 2 Experimental design, materials and methods

### 2.1 Sample collection

The sampling of cattleyak and yak, and preparing of testis from each animal was described in Reference [1].

### 2.2 Protein preparation

Each testicular sample was ground into powder by successively adding liquid nitrogen in a mortar. Then each sample powder was extracted with lysis buffer and the protein was prepared as described in Reference [1].

### 2.3 iTRAQ Labeling and SCX fractionation

Total protein (100 μg) was extracted from each sample solution. After digestion with Trypsin Gold (Promega, Madison, WI, USA), peptides from each sample were dried and processed according to the manufacture׳s protocol for 8-plex iTRAQ reagent (Applied Biosystems). SCX fractionation was performed as description in Reference [1].

### 2.4 LC–ESI-MS/MS analysis based on Triple TOF 5600 and data analysis

The preparation of each SCX fraction, data acquisition and data analysis were performed as description in Reference [1].

In total, 552 DEPs met the criteria for analysis (signal present in all three biological replicates, minimum fold change of ±1.2 or greater and *p*<0.05) by comparing of the testis proteome between cattleyak and yak, in which 206 proteins were up-regulated (Supplementary Table 1) and 346 ones (Supplementary Table 2) were down-regulated in cattleyak with respect to yak.

### 2.5 Gene ontology (GO) analysis of DEPs

GO enrichment analysis firstly maps all DEPs to GO terms in the database (http://www.geneontology.org/), calculating protein numbers for every term. Hypergeometric test was employed to screen significantly enriched GO terms from DEPs by using a strict algorithm as follows:

$$P=1-\overset{m-1}{\underset{i=0}{\sum }}\frac{\left(\underset{i}{M}\right)\left(\underset{n-i}{N-M}\right)}{\left(\underset{n}{N}\right)}$$

*N* denotes the number of all proteins and *n* denotes the number of DEPs in *N*; *M* is the number of all proteins that are annotated to certain GO terms and m denotes the number of DEPs in *M*. The *p*-value is subjected to Bonferroni Correction, with the corrected *p*-value ≤0.05 as a threshold. GO terms fulfilling this condition are defined as significantly enriched GO terms in DEPs.

**Fig. 1:**
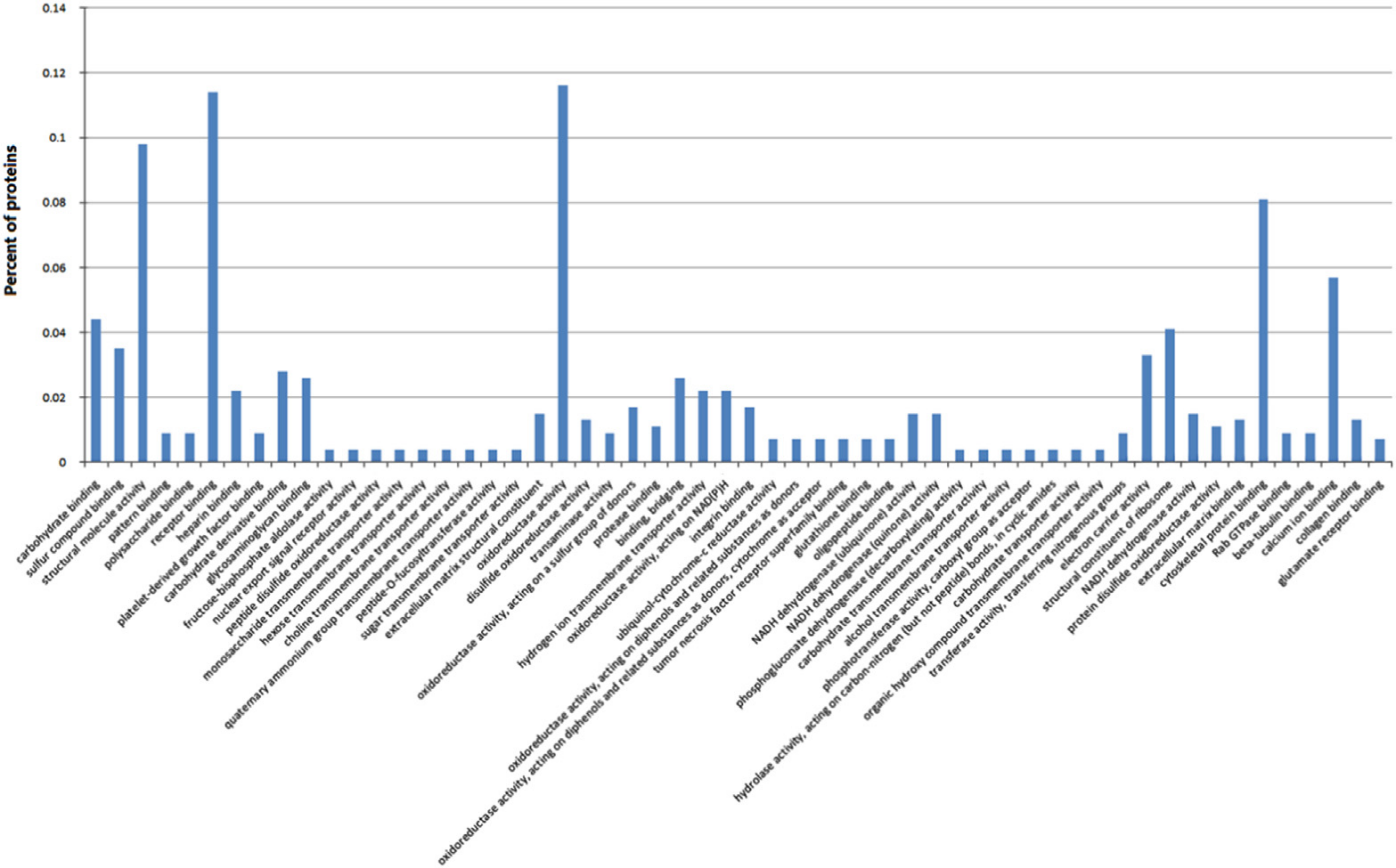
Significant GO terms of differentially expressed proteins on the basis of molecular function. The *x*-axis and *y*-axis correspond to GO terms and percent of genes, respectively. GO terms in each ontological category were ranked according to increased p-value and listed on the *x*-axis from left to right.

All DEPs (552) screened from testis proteome of cattleyak and yak were analyzed by GO according to cellular component, molecular function, and biological process. The 28 significantly enriched GO terms with respect to cellular component were mainly associated with extracellular matrix organization, collagen fibril organization and cytoplasmic part [1]. In contrast, 56 significantly enriched GO terms were mainly were involved in binding, transporter and oxidoreductase activity in molecular function (Fig. 1). Furthermore, 200 GO terms were significantly enriched based on biological process and they were mainly involved in extracellular matrix organization, germ cell development, response to stimulus and metabolic process (Supplementary Fig. 1).

### 2.6 Pathway enrichment analysis of DEPs

KEGG (the major public pathway-related database) [2] is used to identify significantly enriched metabolic pathways or signal transduction pathways in DEPs comparing with certain proteome background. Hypergeometric test was also employed to screen significantly enriched pathways from DEPs by using a strict algorithm as that in GO analysis. Here, *N* denotes the number of all proteins that with KEGG annotation and n denotes the number of DEPs in *N*. *M* is the number of all proteins annotated to specific pathways and m is the number of DEPs in *M*.

**Fig. 2:**
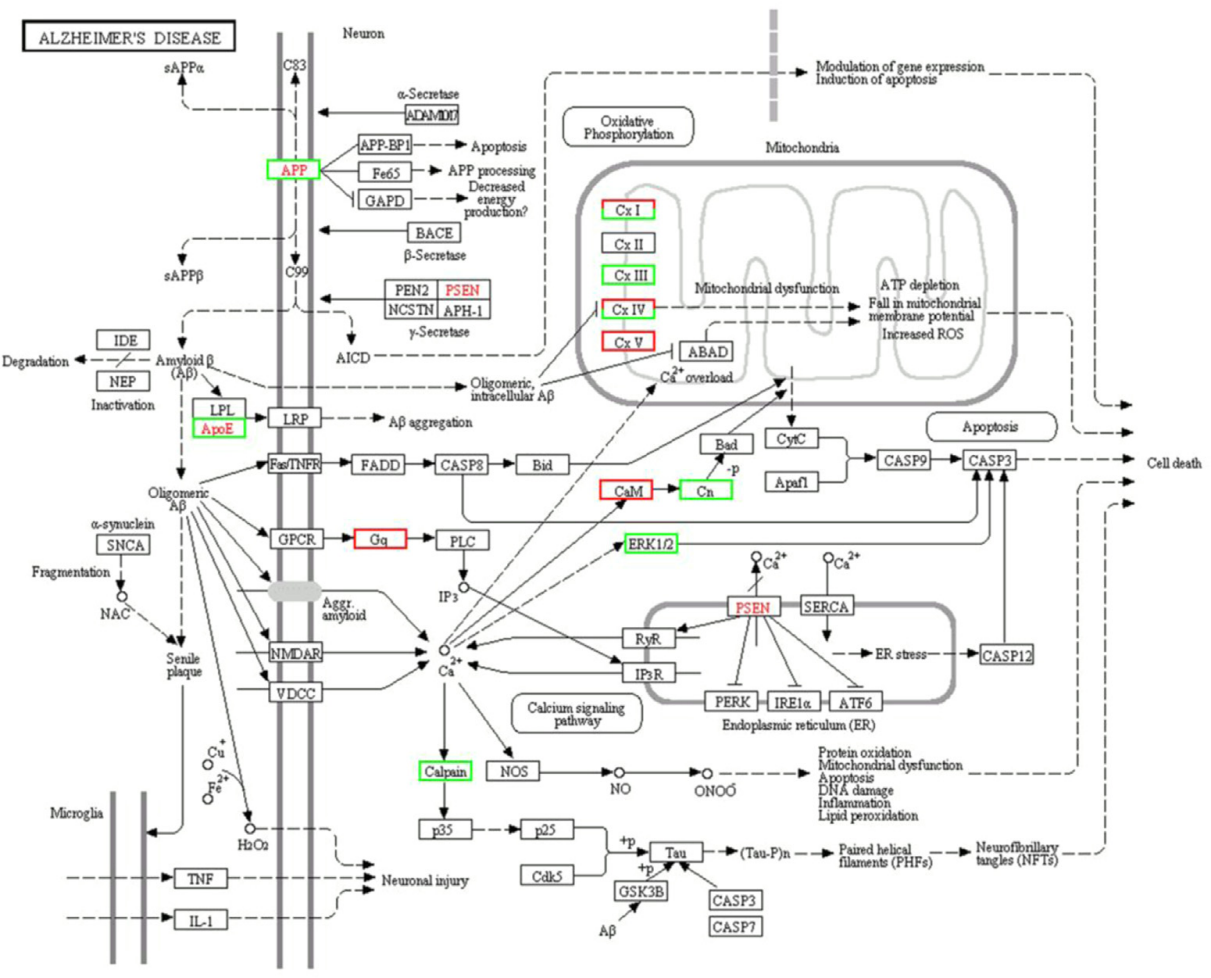
Differentially expressed proteins involved in the pathway of Alzheimer׳s disease. Red-boxed proteins were upregulated and green-boxed proteins were downregulated.

**Fig. 3:**
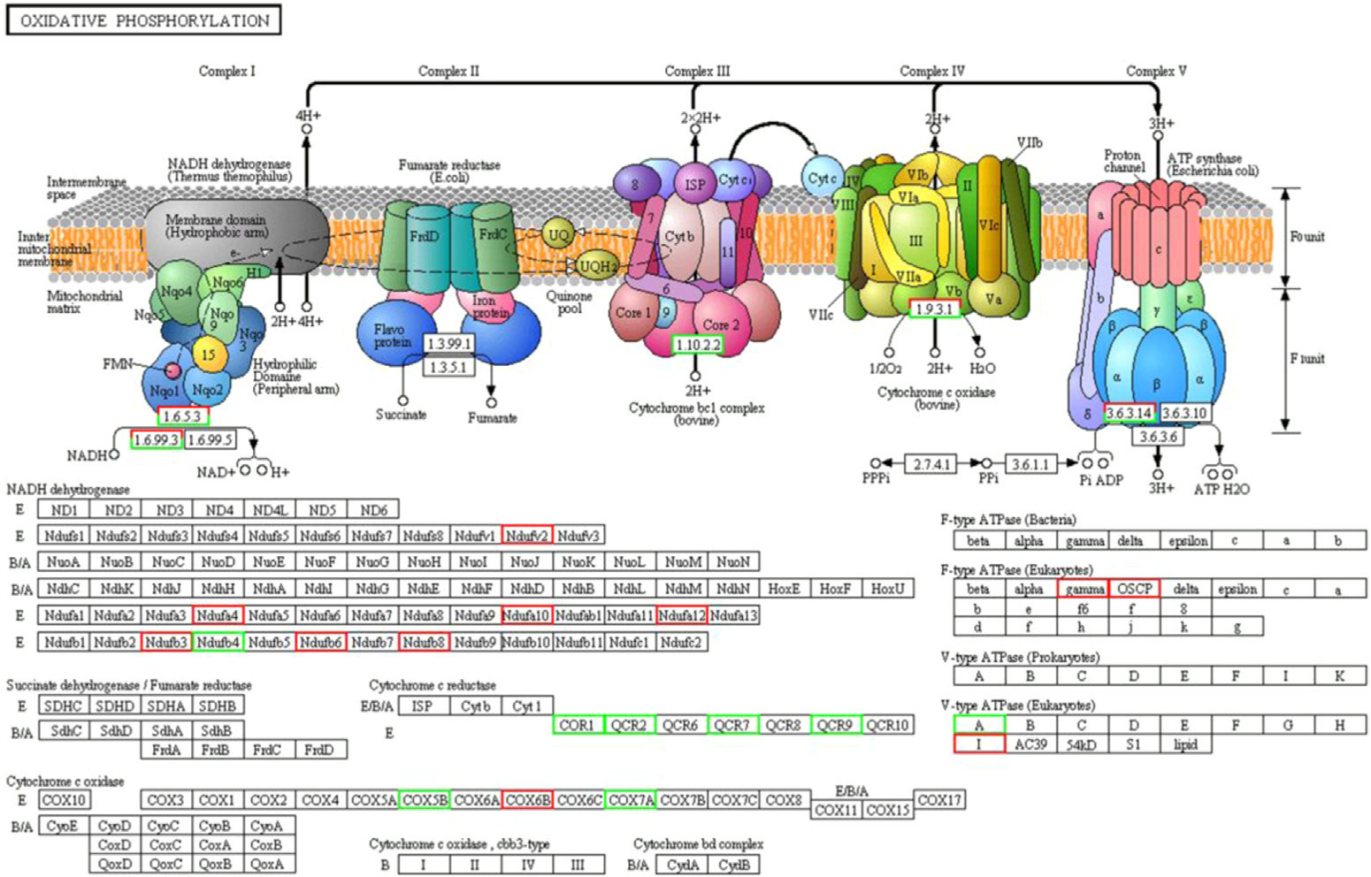
Differentially expressed proteins involved in the pathway of oxidative phosphorylation. Red-boxed proteins were upregulated and green-boxed proteins were downregulated.

In total, 465 DEPs were mapped to the reference pathways in KEGG database and only 15 significantly enriched pathways were obtained (*p*≤0.05), in which ECM-receptor interaction, Protein digestion and absorption, Alzheimer׳s disease, Ribosome and Pentose phosphate pathway were the top listed pathways [1]. Mitochondrial dysfunction involved in such pathways as Alzheimer׳s disease (Fig. 2) and oxidative phosphorylation pathways (Fig. 3) resulted from downregulation of some proteins mitochondrial cytochrome complex may lead to cell death in cattleyak testis.
