# Supplementary material for: Comparative testis proteome dataset between cattleyak and yak
Source: Data Brief. 2016 Jun 3;8:420–5. doi: 10.1016/j.dib.2016.05.071 (PMC4910294; doi:10.1016/j.dib.2016.05.071)
Supplement: Supplementary file 1 — Supplementary material [file mmc1.zip › Supplementary files/Supplemental Figure 1 caption.docx]

Supplemental Fig. 1: Significant GO terms of differentially expressed proteins on the basis of biological process. The x-axis and y-axis correspond to percent of genes and GO terms, respectively. GO terms in each ontological category were ranked according to increased p-value and listed on the y-axis from down to up.
