# Supplementary material for: Comparative testis proteome dataset between cattleyak and yak
Source: Data Brief. 2016 Jun 3;8:420–5. doi: 10.1016/j.dib.2016.05.071 (PMC4910294; doi:10.1016/j.dib.2016.05.071)
Supplement: Supplementary file 1 — Supplementary material [file mmc1.zip › Supplementary files/Supplemental Table 2 caption.docx]

Supplemental Table 2: Proteins down-regulated in cattleyak with respect to yak
