# Supplementary figures and images for: Comparative testis proteome dataset between cattleyak and yak
Source: Data Brief. 2016 Jun 3;8:420–5. doi: 10.1016/j.dib.2016.05.071 (PMC4910294; doi:10.1016/j.dib.2016.05.071)

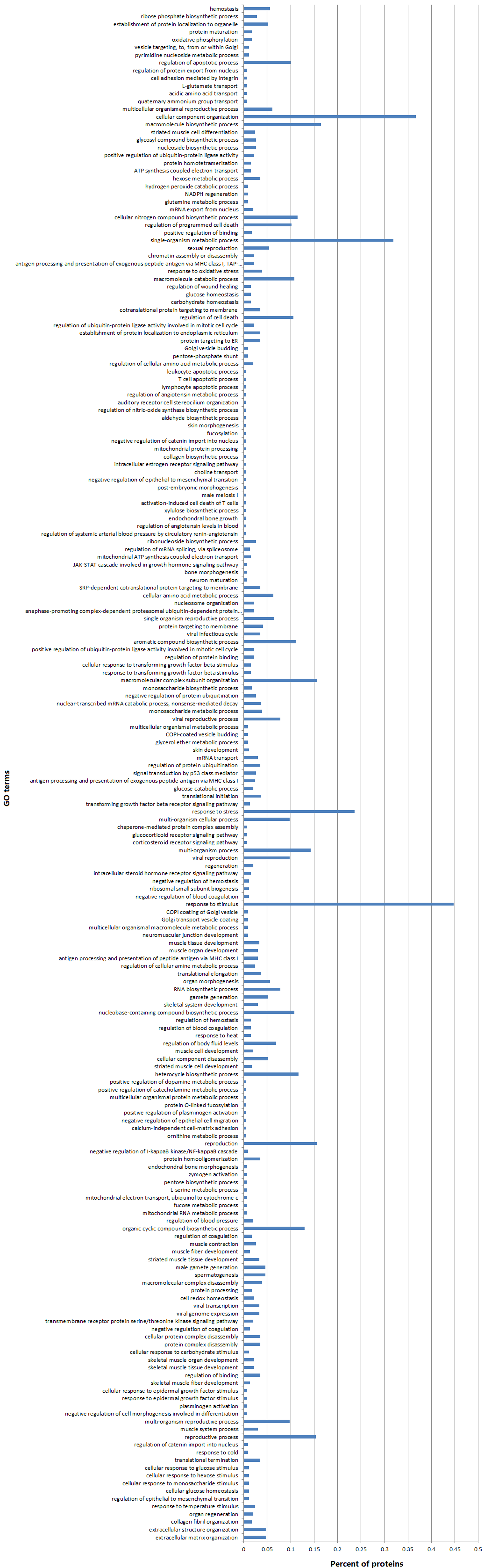

Supplement: Supplementary file 1 — Supplementary material [file mmc1.zip › Supplementary files/Supplementary figure 1.tif]
